# The Association of Delayed Milk Ejection and Milk Production in Dairy Cows Milked by an Automated Milking System

**DOI:** 10.3390/ani15071011

**Published:** 2025-03-31

**Authors:** Matthias Wieland, Heleen ten Have

**Affiliations:** 1 Department of Population Medicine and Diagnostic Sciences, Cornell University, Ithaca, NY 14853, USA; 2 Lely Industries N.V., 3147 PB Maassluis, The Netherlands; htenhave@lely.com

**Keywords:** bovine, bimodal milk flow, milk flow rate, robotic milking

## Abstract

This retrospective cohort study examined the association between delayed milk ejection (DME), defined as bimodal milk flow, and milk yield in dairy cows milked with an automated milking system (AMS). Additionally, we identified risk factors for DME. Using data from a farm milking approximately 1350 cows, we analyzed 689,484 individual milking records and 194,142 daily cow observations over 350 days with generalized linear mixed models. Cows with DME generally had higher daily milk yields, regardless of lactation number. However, first-lactation cows early in lactation and older cows (≥third lactation) late in lactation produced less milk when experiencing DME. In contrast to the higher daily milk yield, cows produced less milk per milking when experiencing delayed milk ejection. However, more frequent milkings contributed to higher daily milk yield, even with more instances of delayed milk ejection. Risk factors for DME included lactation number, stage of lactation, milking frequency, and milking interval. These findings underscore the complexity of DME in AMS and suggest that optimizing individualized milking protocols could improve milk yield efficiency. Understanding the interplay of cow characteristics and milking management may enhance AMS performance and dairy herd productivity.

## Simple Summary

Some dairy cows experience delayed milk ejection, meaning their milk flow is slower or interrupted during milking. In conventional milking systems, this is usually linked to lower milk production. In this study, we looked at how delayed milk ejection affects milk yield in cows milked by robots. Interestingly, the impact varied depending on the cow’s age and stage of lactation. For most of the lactation period, delayed milk ejection was linked to higher milk yield. However, younger cows at the start of their first lactation and older cows late in their lactation produced less milk when they experienced delayed milk ejection. When a cow had delayed milk ejection, she produced less milk during that particular milking. However, because cows with more milkings tended to have a higher overall daily milk yield, their total milk production remained high even if some milkings were affected by delayed milk ejection. These findings suggest that when evaluating how delayed milk ejection affects milk production, it is important to consider the cow’s age, lactation stage, and the entire lactation period.

## 1. Introduction

The analysis of milk flow curves provides valuable insights for improving milking efficiency and enhancing udder health by optimizing milking equipment and protocols to meet the cow’s physiological requirements [1]. Tančin et al. [2] identified four phases of milk flow curve intensity: (1) incline, (2) plateau, (3) decline, and (4) overmilking phase. The shape of the milk flow curve during the first two minutes of milking has been linked to the effectiveness of premilking udder preparation [3]. Tactile teat stimulation during this preparation is crucial for activating the milk ejection reflex, which releases the alveolar milk fraction into the gland cistern [4]. A milk flow curve from a cow receiving adequate tactile stimulation and proper timing of milking unit attachment (i.e., preparation lag time) typically shows a short (~15 s) and steep incline that transitions seamlessly into the plateau phase [5]. Conversely, insufficient stimulation, inadequate preparation lag time, or both can cause delayed milk ejection (DME) [4].

Delayed milk ejection (DME) often manifests as bimodal milk flow. A milk flow curve is classified as bimodal when an increasing milk flow rate is followed by a decreasing flow rate within the first two minutes of milking [2], typically due to the removal of the cisternal milk fraction before the alveolar milk enters the gland cistern [4]. However, DME can also occur without bimodal milk flow, particularly when only small amounts of cisternal milk are available, such as after short milking intervals [6]. Delayed milk ejection has been linked to decreased milking efficiency [7], reduced milk yield [8], and impaired teat and udder health [7,9,10]. Recent studies, including work from our group, show that cows with DME produce 1.3 to 3.1 kg less milk per milking session compared to cows without DME [8,11].

Most research on DME in dairy cows has been conducted using conventional milking systems [8,11,12]. By contrast, knowledge about DME and its possible sequelae in dairy cows milked by automated milking systems is scarce. This leaves large knowledge gaps in what is likely one of the most important factors for sustainable automated milking systems. The primary objective of the study described herein was to investigate the association of DME with milk yield in dairy cows milked by an automated milking system. We hypothesized that DME was associated with decreased milk yield in dairy cows milked by an automated milking system.

Multiple research groups have investigated the risk factors for bimodality and identified breed [13], stage of lactation [1], the degree of udder filling [9], chronic mastitis [10], health and management events [14], and teat traits such as teat shape [15] as factors associated with the occurrence of bimodality. However, these studies have largely focused on conventional milking systems. Thus, knowledge of risk factors specific to cows milked by automated systems remains scarce. Our second objective was to investigate the risk factors for bimodality in cows milked by an automated milking system.

## 2. Materials and Methods

Data for this retrospective cohort study were provided by Lely Industries N.V. (Maassluis, The Netherlands). The data were retrieved from one Canadian dairy herd with automated milking systems. Cows (n ≈ 1350) were housed year-round in 4-row free-stall pens bedded with chopped straw with hydrated lime two (old facilities) to seven (new facilities) times per week. Cows were fed a partial mixed ration that was supplemented with concentrate (amount per visit/customized for each cow based on milk production and lactation stage) provided in the automated milking system (Astronaut A5, Lely Industries N.V., Maassluis, The Netherlands). Cows were milked with 20 automated milking systems in a hybrid flow traffic system. Milking permission was based on stage of lactation (5.4-, 3.2-, and 1.8-times daily) for early- (<80 DIM), mid- (80 DIM to 22 days before expected dry-off), and late lactation (21 days before expected dry-off date) cows, respectively, and expected milk production with a minimum milking interval of 4 h since the last successful milking. Cows that were 21 days prior to the dedicated dry-off were subjected to a minimum milking interval of 10.8 h. The premilking teat preparation protocol was as follows: upon cow identification, each teat was cleaned with rotating brushes for a duration of two to three seconds. After the first round of teat cleaning, the brush was cleaned with a solution based on peracetic acid and subsequently dried. Then, the teats were cleaned again with the rotating brushes for a duration of one second per teat. After teat preparation, the brushes were cleaned again for the next milking. After teat sanitization, teat cups were attached with one of the hind quarters being attached first. This protocol resulted in a stimulation duration (duration of tactile stimulation via the teat brush) of twelve to 16 s and a preparation lag time (time period between first tactile stimulus and attachment of the first teat cup) of 70 s.

The daily milk production (mean ± standard deviation, SD) was 42.0 ± 10.7 kg/day, and the mean (±SD) SCC was 89,000 ± 116,000 cells/mL. Data on cow characteristics included lactation number and stage of lactation (days in milk; DIM) and were obtained from the dairy management software program (Horizon, Lely Industries N.V., The Netherlands). Data on milking characteristics were obtained at each milking session with electronic on-farm milk meters and recorded using the dairy farm management software and included cow identification, lactation number, DIM, date and time of milking, total milk yield harvested from all lactating quarters (kg/milking session), presence or absence of a bimodal milk flow curve at the quarter-level (present or absent), failed milking attempt (present or absent, i.e., no successful connection to the teats, no detection of milk flow for any of the quarters that should be milked, or abortion of the robotic arm), and milking interval (time elapsed since last milking). The detection of a bimodal milk flow curve at the quarter-level was performed by means of indirect milk flow measurements using electrical conductivity and based on the principal outlined by Tančin et al. [2]. That is, a bimodal milk flow curve is characterized by an initial increase in milk flow rate, followed by a subsequent decrease within the first two minutes of milking [2]. Accordingly, a semi-supervised model was trained with manually labeled cow milking observations to automatically detect bimodality.

### 2.1. Analytical Approach

We maintained data in Excel (Microsoft Excel, 2022 version, Microsoft Corp., Redmond, WA, USA) and JMP (JMP Pro 17, SAS Institute Inc., Cary, NC, USA). Statistical analyses were performed with R [16].

### 2.2. Data Processing

The original data file included data from 13 May 2022 to 21 August 2023 and consisted of 1,654,178 individual milking observations from 2505 cows contributing 3519 cow lactations (1491 cows contributed one lactation, 1014 cows contributed two lactations). To give each cow an equal weight to the analyses, only one lactation from each cow was included. For this purpose, we removed the shorter lactation in each of the 1014 cases. Further, data from cows that had their calving date before 13 May 2022, milking observations that were recorded after 350 DIM, and milking observations with a ‘failed milking flag’ were excluded.

For further analyses, the data were investigated for missing values and the following variables were created: daily milk yield (calculated by summing the total milk yields from all milking sessions observed throughout the day), number of milking observations per day (calculated as the sum of milking observations documented throughout the day), bimodal cow-level milking observation (defined as present if one or more of the quarter-level milk flow curves were detected as bimodal by means of the quarter-level ‘bimodality prediction flag’), number of bimodal milking observations per day (calculated as the sum of bimodal cow-level milking observations throughout the day), percentage of bimodal milk flow curves per day (calculated as number of bimodal milking observations per day divided by number of milking observations per day), and bimodal-3 (categorical variable generated from the percentage of bimodal milk flow curves per day as follows: level 1, 0%; level 2, 1–50%; level 3, >50%). The three levels for bimodal-3 were chosen based on the frequency distribution of bimodality. Additionally, we selected a threshold value of 50% as it represents the majority of daily milking events, providing a biologically and statistically meaningful cutoff for identifying cows consistently exhibiting bimodality within a given day.

### 2.3. Data Analyses

We tested our hypothesis that DME would be associated with milk production in two approaches. In a first step, we estimated differences in total milk yield among individual cow milking observations with and without bimodality. In a second step we investigated the association of bimodal-3 with daily milk yield.

To study differences in total milk yield (kg/milking session) among individual cow milking observations with and without bimodality (Model I), we fitted a general linear mixed model with the ‘lme4’ package [17] in R. The outcome variable of interest was total milk yield (kg/milking session) and included as dependent variable. The independent variable of interest was the binary variable presence or absence of bimodality. Parity (1st, 2nd, 3rd or greater), stage of lactation (1–100, 101–200, 201–350 days in milk), number of milkings per day as a categorical variable (1–6), and milking interval as a continuous variable (hours) were included as covariates. We used three levels for the categorization of stage of lactation in accordance with previous research investigating the association of DME with milk yield [11]. To account for the clustered structure of the data, a random effect for cow was included. Estimated marginal means were calculated with the ‘emmeans’ package [18] in R. To control for the familywise error rate when comparing a family of estimates, we applied Tukey–Kramer’s post hoc test. Significance was declared at *p* < 0.05. We inspected residual plots versus corresponding predicted values and examined quantile–quantile residual plots to assess whether the assumptions of homoscedasticity and normality of residuals were met.

To study the association of bimodal-3 and daily milk yield, we fitted four separate general linear mixed models with the ‘lme4’ package [17] in R. The following steps were consistent for all four models: Cow was included as random effect to account for the clustered structure of the data. Daily milk yield (kg/day) was the outcome variable of interest and included as dependent variable. Bimodal-3 was the explanatory variable of interest and included as independent variable. Estimated marginal means (EMM) were calculated with the ‘emmeans’ package [18] in R. Significance was declared at *p* < 0.05. The assumptions of homoscedasticity and normality of residuals were assessed by the inspection of residual plots versus corresponding predicted values and the examination of quantile-quantile residual plots.

In Model II, parity (1st, 2nd, and ≥3rd lactation) and stage of lactation (1–60, 61–120, 121–180, 181–240, 241–300, 301–350 days in milk) were included as covariates. We selected six levels to categorize the state of lactation after an initial data screening indicated that daily milk yield might vary among cows in these different categories. The 2-way interactions between bimodal-3 and parity and between bimodal-3 and stage of lactation, as well as the 3-way interaction between bimodal-3, parity, and stage of lactation were also included. Tukey–Kramer’s post hoc test was used to control for the experimental error rate.

In Models III–V, we analyzed each parity group in a separate model. Stage of lactation (DIM in 7-day increments) was included, as well as the 2-way interaction between bimodal-3 and stage of lactation. We used 7-day increments to define the stage of lactation to facilitate the graphical representation of the results.

To investigate risk factors for the occurrence of bimodality, we fitted a generalized linear mixed model with a logit link and a binomial distribution with the ‘lme4’ package [17] in R. Cow was included as a random effect to account for the clustered structure of the data. The binary variable presence or absence of bimodality was included as the dependent variable. Parity (1st, 2nd, and 3rd or greater), stage of lactation (1–100, 101–200, and 201–350 days in milk), number of milkings as a categorical variable (1–6), and milking interval as a continuous variable (hours) were included as independent variables. We evaluated the final model’s predictive ability by calculating a receiver operating characteristic curve with the ‘pROC’ package [19].

## 3. Results

### 3.1. Study Population

After exclusion of the second (shorter) lactation from cows contributing two lactations from the original data set, exclusion of data from cows that had their calving date before 13 May 2022, milking observations that were recorded after 350 DIM, and observations with a ‘failed milking flag’, we obtained a total of 922,192 individual milking observations from 1687 cows. These resulted in a total of 311,712 daily cow observations.

For a total of 232,708/922,192 (25.2%) milking observations, data on the presence or absence of bimodality was missing for at least one quarter-level milking observation. The exclusion of these observations resulted in 689,484 individual milking observations from 1580 cows. A total of 101,026 (14.7%) individual milking observations showed a bimodal milk flow curve.

The exclusion of 117,570 daily cow observations for which one or more individual milking observations had a missing value, resulted in a total of 194,142 daily cow observations from 1573 cows that were available for the final analyses. These cows were in their 1st (1031, 65.5%), 2nd (260, 16.5%), and 3rd or greater (282, 17.9%) lactation. The average (mean ± SD) number of milkings per day was 3.0 ± 0.8, ranging from 1 to 6. The average (mean ± SD) percentage of bimodal milk flow curves per day was 12.7 ± 24.7%. The frequency distribution of bimodal-3 was level 1 (0%), 144,214 (74.3%); level 2 (1–50%), 34,953 (18.0%); and level 3 (>50%), 14,975 (7.7%). Table 1 shows the average number of milkings per day and the frequency distribution of bimodal-3 stratified by parity and stage of lactation. The average (mean ± SD) daily milk yield was 38.9 ± 12.7 kg/day. Table 2 shows the average daily milk yield stratified by bimodal-3, parity, and stage of lactation.

### 3.2. Model I

The final model I included parity (*p* < 0.0001), stage of lactation (*p* < 0.0001), number of milking per day (*p* < 0.0001), milking interval (*p* < 0.0001), and the presence or absence of a bimodal milk flow curve (*p* < 0.0001). The EMM (95% confidence intervals, 95% CI) among cows with different lactation numbers were parity 1, 9.7 (9.6–9.8); parity 2, 13.9 (13.7–14.1); and parity 3 or greater, 14.8 (14.6–15.0) kg. The Tukey–Kramer post hoc test revealed differences among all groups (*p* < 0.0001). The EMM (95% CI) for cows in different stages of lactation were 1–100 DIM, 12.6 (12.4–12.7); 101–200 DIM, 13.2 (13.1–13.4); and 201–350 DIM, 12.6 (12.5–12.8) kg and, based on the Tukey–Kramer post hoc test, were different among groups (*p* < 0.0001). The EMM (95% CI) for different numbers of milking per day were 1, 13.3 (13.2–13.4); 2, 13.3 (13.2–13.4); 3, 12.9 (12.8–13.0); 4, 12.6 (12.5–12.7); 5, 12.4 (12.3–12.6); and 6, 12.3 (12.0–12.7) kg. The Tukey–Kramer post hoc test revealed differences between all groups (*p* < 0.0001), except for 1 versus 2, 4 versus 6, and 5 versus 6 (*p* ≥ 0.75). A 1 h increase in the milking interval increased the milk yield by 0.4 kg. Controlling for the effects of parity, stage of lactation, and number of milkings per day, the EMM (95% CI) were 12.2 (12.1–12.4) kg for cow milking observations with a bimodal milk flow curve and 13.4 (13.3–13.5) kg for milking observations without a bimodal milk flow curve. The assumptions of homoscedasticity and normality of residuals were met.

### 3.3. Model II

The final model II included parity (*p* < 0.0001), stage of lactation (*p* < 0.0001), bimodal-3 (*p* < 0.0001), their 2-way interactions (*p* < 0.0001), and their 3-way interaction (*p* < 0.0001). Table 3 shows the EMM and 95% CI for the 3-way interaction. Based on the inspection of residual plots versus corresponding predicted values and the examination of quantile-quantile residual plots, the assumptions of homoscedasticity and normality of residuals were met.

### 3.4. Models III–V

**Figure 1:**
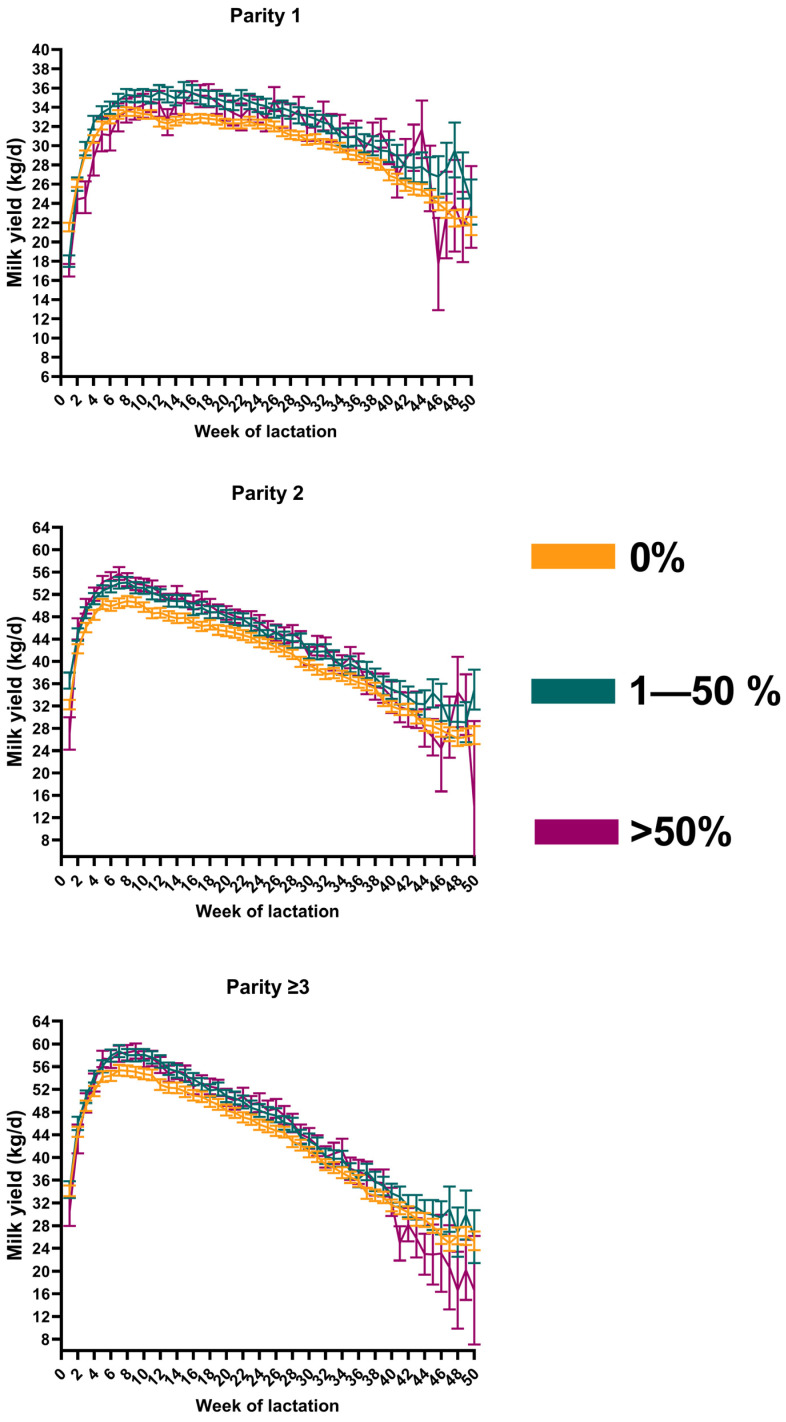
Estimated marginal means showing the average daily milk yield (kg/d) over the course of 50 weeks from 194,142 daily cow observations of 1573 dairy cows exhibiting a bimodal milk flow curve in 0, 1–50%, and >50% of milking observations per day stratified by parity. Error bars show 95% confidence intervals.

The final models III–V included stage of lactation (in 7-day increments, *p* < 0.0001), bimodal-3 (*p* < 0.0001), and their interaction (*p* < 0.0001). Figure 1 shows the EMM (95% CI) for daily milk yield for cows in parity 1 (A), 2 (B), and 3 or greater (C). The assumptions of homoscedasticity and normality of residuals were met.

### 3.5. Risk Factors for Bimodality

The final generalized linear mixed model included parity (*p* < 0.0001), stage of lactation (*p* < 0.0001), number of milkings per day (*p* < 0.0001), and milking interval (*p* < 0.0001). Compared with cows in parity 3 and greater, the odds (odds ratio, OR; 95% CI) of a bimodal milk flow curve was 0.31 (0.26–0.37) for cows in parity 1 and 1.23 (0.98–1.55) for cows in parity 2. Early- (1–100 DIM) and mid-lactation (101–200 DIM) cows had lower odds of bimodality compared to late-lactation (201–350 DIM) cows (OR, 95% CI; 1–100 DIM, 0.59 (0.58–0.61); 101–200 DIM, 0.87 (0.85–0.89)). Compared with one milking per day, the odds of bimodality were 0.85 (0.76–0.95) for two milkings, 0.83 (0.75–0.93) for three milkings, 1.12 (1.00–1.25) for four milkings, 1.84 (1.63–2.07) for five milkings, and 2.81 (2.28–3.46) for six milkings per day. A 1 h increase in the milking interval decreased the odds of bimodality (OR, 95% CI; 0.72 (0.72–0.73). The area under the receiver operator receiver operating characteristic curve was 0.86.

## 4. Discussion

The primary objective of the study described herein was to investigate the association of DME with milk production in dairy cows milked by an automated milking system. For this purpose, we analyzed data from a single dairy farm using 20 automated milking systems and used three different approaches to evaluate if DME was associated with milk yield.

The analysis of the individual milking observations revealed that bimodality was negatively associated with milk yield. Observations with a bimodal milk flow curve yielded 1.2 kg less milk compared to those without. These results are consistent with those reported previously from studies conducted in conventional milking systems [8,11,20]. Erskine et al. [8] reported that milk yield decreased by 1.8 to 3.1 kg per milking session for milking observations with DME. Wieland et al. [11] demonstrated that milk yield was reduced by 1.3 kg per milking session for milking observations with a bimodal milk flow curve. The results of a recent meta-analysis revealed that DME can reduce milk yield by 1.55 kg per milking session [20].

As discussed previously [8,11,20], the milk yield reduction in bimodal observations could be attributed to decreased intracisternal pressure, reduced teat barrel diameter, and a poor seal between the teat and milking liner [21]. These conditions may elevate mouthpiece chamber vacuum, leading to tighter seals at the teat base, congestion, reduced teat canal cross-section, and ultimately hampered milk flow [21,22].

Delayed milk ejection has been described as a function of the degree of udder filling [3,9] and the duration of the milking interval [9] with lower degrees of udder filling and shorter milking intervals leading to DME. Delayed milk ejection has also been associated with shorter duration of premilking teat stimulation [23], inadequate preparation lag time (time period between premilking stimulation and milking unit attachment) [24], or a combination of both [4]. Kaskous and Bruckmaier [3] suggested that a stimulation duration of 15 s prior to a latency period of 45 s resulting is necessary to achieve adequate milk ejection in mid-lactation cows. For cows with very low udder filling, the researchers suggested a stimulation time of 30 s followed by a latency period of 60 s [3].

Similarly, Weiss and Bruckmaier recommended a premilking stimulation duration of 20 to 90 s based on the degree of udder filling [25]. For automated milking systems, with variable and sometimes extremely short milking intervals, Bruckmaier and Hilger [9] recommended that the duration of premilking teat stimulation should be adapted to the expected milk yield at each individual milking session.

One may, therefore, argue that the DME and the associated decrease in milk yield documented in the current study could be alleviated through the application of adequate premilking teat stimulation tailored to each individual cow milking observation. However, this theory remains speculative. As discussed by Dahl and Wieland [20], this theory is mainly based on observational studies. They reported that only one out of six interventional studies, the study described by Mayer et al. [26], provided evidence that the application of premilking teat stimulation would lead to a decrease in bimodal milk flow curves and an increased milk yield. In contrast, Bruckmaier and Blum [7] found that cows that did not receive premilking stimulation exhibited bimodality and increased milking duration, but no significant decrease in milk yield. This is consistent with data from our own group showing that a short stimulation duration or preparation lag time led to higher odds of bimodality and increased milking unit on time but did not negatively affect milk yield [15,24]. Future studies under controlled settings are necessary to investigate if an extended premilking stimulation regimen can reduce the occurrence of DME and increase the milk production of cows exhibiting DME milked by an automated milking system.

Compared to the individual milking observations, the analysis of daily cow observations (Models II–V) over a 350-day lactation period produced seemingly conflicting results. We found that bimodality was associated with higher milk yield for most of the lactation period among cows of all parity groups with differences ranging from 1.5 to 7.4 kg milk per day. To the best of our knowledge, this is the first study investigating the association of DME with milk yield over the course of a lactation. This unexpected finding may be explained by the benefits of higher milking frequency outweighing the negative effects of DME. In this study, cows accessed the automated milking system up to six times daily, with higher frequencies correlating with increased milk yield [27,28,29] despite a higher occurrence of bimodality.

First-lactation cows during their first 60 DIM exhibiting bimodality in over 50% of observations had lower daily yields. This is likely due to disturbed milk ejection caused by central inhibition of oxytocin release in unfamiliar surroundings [30,31]. For first lactation animals at the beginning of their lactation, the milking station undoubtedly represents unfamiliar surroundings.

Late-lactation cows (301–350 DIM) in parity three or greater also experienced lower yields with frequent bimodality, likely due to inadequate stimulation relative to udder filling. Previous research showed that a short premilking stimulation duration in late-lactation cows with lower udder filling increases the occurrence of bimodal milk flow curves [3,25]. They suggested increasing the duration of the premilking teat stimulation and the subsequent latency period for late-lactation cows with low udder filling [3,25]. Adjusting premilking stimulation regimens for these groups may mitigate bimodality and improve milk yields.

The second objective of our study was to investigate risk factors for bimodality in cows milked by an automated milking system. First-lactation cows had lower odds of bimodality compared to cows in parity three or greater, consistent with some prior studies [23] but conflicting with others [1,14]. We believe that differences in study populations and diagnostic techniques help explain the discrepancies among studies. Early- (1–100 DIM) and mid-lactation (101–200 DIM) cows had lower odds compared to those at 201–350 DIM. These results are in accordance with those in the aforementioned studies [1,14] but contrast those reported by Erskine et al. [8] who found no differences in DME occurrence among different categories of stage of lactation. Increased milking frequency (five or more times daily) raised bimodality odds, while a one-hour increase in interval reduced the odds by 38%, reflecting findings from previous research [9].

Our study had some limitations. This study was conducted on a single farm with a high proportion of first-lactation cows, limiting generalizability. Before these results can be extrapolated, this study needs to be replicated on other farms with different study populations, (automated) milking systems, management systems, and regions. Second, the exclusion of 25.2% of individual milking observations and 37.7% of daily cow observations due to missing values could have led to selection bias. Third, our case definition for bimodality was based on a customary machine learning algorithm. Although we were able to identify biologically plausible associations, the biological relevance of the applied case definition still needs to be confirmed by further studies. Similarly, the selection of the three categories for bimodal-3 was based on the frequency distribution, combined with our impression that a threshold of 50% reflects the majority of daily milking events, providing a biologically meaningful cutoff for identifying cows that consistently exhibit bimodality within a given day. However, this case definition could be adjusted depending on individual farm goals. Last, as an observational study, causation cannot be established.

There are numerous opportunities for future research. First, as noted above, further studies are needed to determine the most effective and discriminatory case definition for bimodality. Such research should not only focus on milk production but also consider the reported negative effects of bimodality on teat tissue condition, udder health, and overall animal well-being. Second, future research should investigate whether extended premilking stimulation regimens can reduce bimodality and enhance milk yield across a variety of farm conditions. These studies should also account for differences in parity and stage of lactation. Lastly, future research should further investigate the optimal milking frequency for cows milked by automated milking systems. Such studies should consider factors such as udder filling, udder storage capacity, and the occurrence of bimodality to help determine the optimal number of milkings per day.

## 5. Conclusions

The analysis of individual milkings showed that DME was associated with lower milk yield per session. We attributed this to a discrepancy between udder filling, milking interval, and premilking teat stimulation. Lower udder filling and shorter intervals likely increased the occurrence of DME, while inadequate premilking stimulation and teat-vacuum-liner interactions may have further disrupted milk flow, ultimately hindering milk ejection efficiency. However, further research is needed to validate this hypothesis.

The analysis of daily observations over a 350-day lactation showed that, for most of the lactation period, cows exhibiting DME produced higher daily milk yields regardless of age. This increase was attributed to higher milking frequency in high-producing cows, which appeared to offset the negative effects of DME, as cows in this study accessed the automated milking system up to six times daily, with greater frequency correlating with increased milk yield despite a higher occurrence of bimodality. In contrast, first-lactation cows in early lactation and multiparous cows (third lactation or greater) in late lactation exhibited reduced milk yields when DME was present in more than 50% of their daily milkings. We believe the lower yields in first-lactation cows may result from disrupted milk ejection as they adjust to the unfamiliar milking environment, whereas the declines in late-lactation multiparous cows were likely due to inadequate stimulation relative to udder filling, leading to impaired milk ejection.

These seemingly conflicting results highlight the need to consider the relationship between DME and milking performance in an automated milking system in the context of parity, lactation stage, lactation period, and milking interval. Further research is required to determine whether DME can be leveraged to develop individualized milking protocols that optimize performance, cow health, and profitability in automated milking systems.

## Figures and Tables

**Table 1:** Average (mean ± standard deviation) number of milkings per day and frequency distribution (%) of the percentage of bimodal milk flow curves per day (bimodal-3) from 194,142 cow observations of 1573 dairy cows stratified by parity and stage of lactation (n = number of observations for each group).

	n	Number of Milkings	Bimodal-3		
			0%	1–50%	>50%
Parity 1					
1–100 DIM	48,333	3.1 ± 0.8	84.5	12.2	3.2
101–200 DIM	29,288	2.9 ± 0.8	81.5	13.7	4.7
>200 DIM	22,455	2.5 ± 0.8	83.6	12.2	4.2
Parity 2					
1–100 DIM	17,483	3.5 ± 0.8	56.4	27.3	16.3
101–200 DIM	15,910	3.0 ± 0.7	62.6	22.5	15.0
>200 DIM	11,335	2.4 ± 0.7	75.0	16.5	8.5
Parity ≥3					
1–100 DIM	18,804	3.5 ± 0.8	60.7	28.5	10.8
101–200 DIM	17,945	3.0 ± 0.8	64.2	25.2	10.7
>200 DIM	12,589	2.3 ± 0.7	74.7	17.7	7.6

**Table 2:** Average (mean ± standard deviation) daily milk yield (kg) from 194,142 daily cow observations of 1573 dairy cows stratified by parity, stage of lactation, and the percentage of bimodal milk flow curves per day (bimodal-3).

	Bimodal-3		
	0%	1–50%	>50%
Parity 1			
1–100 DIM	31.4 ± 8.7	34.5 ± 9.5	30.1 ± 11.8
101–200 DIM	33.6 ± 8.2	39.1 ± 7.7	39.2 ± 7.4
>200 DIM	30.2 ± 8.7	35.1 ± 8.1	35.1 ± 8.5
Parity 2			
1–100 DIM	46.0 ± 10.4	52.1 ± 9.5	53.7 ± 9.6
101–200 DIM	43.2 ± 9.3	48.5 ± 9.5	50.1 ± 9.5
>200 DIM	34.7 ± 10.7	40.0 ± 10.0	40.9 ± 11.1
Parity ≥3			
1–100 DIM	51.3 ± 11.5	55.6 ± 11.2	55.6 ± 12.8
101–200 DIM	47.0 ± 10.6	51.1 ± 10.6	51.6 ± 10.6
>200 DIM	35.1 ± 11.4	38.1 ± 10.7	39.2 ± 13.8

**Table 3:** Estimated marginal means and 95% confidence intervals showing the average daily milk yield (kg/d) for cows exhibiting a bimodal milk flow curve in 0, 1–50%, and >50% (bimodal-3) of milking observations per day stratified by parity and stage of lactation. ^a–c^ Groups with different superscript letters in the same row differ at a level of *p* < 0.05 in the Tukey–Kramer post hoc test.

		Bimodal-3	
	0%	1–50%	>50%
Parity 1			
1–60 DIM	29.1 (28.7–29.5) ^a^	29.6 (29.1–30.1) ^b^	23.6 (23.0–24.3) ^c^
61–120 DIM	31.1 (30.7–31.6) ^a^	33.7 (33.2–34.2) ^b^	32.8 (32.2–33.5) ^c^
121–180 DIM	30.7 (30.3–31.1) ^a^	33.0 (32.5–33.5) ^b^	32.2 (31.5–32.9) ^c^
181–240 DIM	28.9 (28.5–29.4) ^a^	31.2 (30.6–31.7) ^b^	30.9 (30.2–31.7) ^b^
241–300 DIM	26.0 (25.5–26.4) ^a^	28.2 (27.6–28.8) ^b^	28.7 (27.9–29.6) ^b^
301–350 DIM	22.0 (21.5–22.5) ^a^	25.5 (24.5–26.6) ^b^	23.7 (21.8–25.5) ^ab^
Parity 2			
1–60 DIM	45.5 (44.6–46.3) ^a^	50.6 (49.8–51.5) ^b^	52.9 (52.0–53.8) ^c^
61–120 DIM	47.2 (46.4–48.1) ^a^	50.6 (49.7–51.4) ^b^	51.9 (51.0–52.7) ^c^
121–180 DIM	43.8 (43.0–44.6) ^a^	46.8 (46.0–47.7) ^b^	47.9 (47.0–48.8) ^c^
181–240 DIM	38.5 (37.6–39.3) ^a^	41.9 (41.0–42.8) ^b^	42.7 (41.7–43.7) ^c^
241–300 DIM	32.7 (31.9–33.5) ^a^	36.3 (35.4–37.3) ^b^	36.0 (34.9–37.2) ^b^
301–350 DIM	27.0 (26.1–27.9) ^a^	32.0 (30.6–33.4) ^b^	28.2 (26.3–30.1) ^a^
Parity ≥3			
1–60 DIM	49.8 (49.0–50.6) ^a^	54.1 (53.3–54.9) ^b^	54.9 (54.0–55.8) ^c^
61–120 DIM	51.9 (51.2–52.7) ^a^	55.5 (54.7–56.3) ^b^	55.3 (54.4–56.1) ^b^
121–180 DIM	46.7 (45.9–47.5) ^a^	49.6 (48.7–50.4) ^b^	50.5 (49.7–51.4) ^c^
181–240 DIM	40.0 (39.3–40.8) ^a^	42.7 (41.8–43.5) ^b^	43.5 (42.6–44.5) ^c^
241–300 DIM	32.2 (31.4–33.0) ^a^	35.2 (34.3–36.1) ^b^	34.3 (33.3–35.4) ^b^
301–350 DIM	26.2 (25.4–27.1) ^a^	29.3 (27.9–30.6) ^b^	21.7 (19.6–23.7) ^c^

## Data Availability

The original contributions presented in this study are included in the article. Further inquiries can be directed to the corresponding author.

## Abbreviations

The following abbreviations are used in this manuscript:

DME	Delayed milk ejection
DIM	Days in milk
EMM	Estimated marginal means
SCC	Somatic cell count
